# Soil mite communities (Acari: Mesostigmata, Oribatida) as bioindicators for environmental conditions from polluted soils

**DOI:** 10.1038/s41598-019-56700-8

**Published:** 2019-12-27

**Authors:** Minodora Manu, Viorica Honciuc, Aurora Neagoe, Raluca Ioana Băncilă, Virgil Iordache, Marilena Onete

**Affiliations:** 1 Romanian Academy, Institute of Biology Bucharest, Department of Ecology, Taxonomy and Nature Conservation, street Splaiul Independenţei, no. 296, Bucharest, Romania; 2 Research Centre for Ecological Services (CESEC), Faculty of Biology, University of Bucharest, Bucharest, Romania; 3 University Ovidius Constanţa, Faculty of Natural Sciences, Al. Universităţii, corp B, Constanţa, Romania; 4 Romanian Academy, “Emil Racoviţă” Institute of Speleology, Department of Biospeleology and Soil Edaphobiology, 13 Septembrie Road, No. 13, 050711 Bucharest, Romania

**Keywords:** Population dynamics, Restoration ecology

## Abstract

An anthropic ecosystem from Romania was investigated from acarological, vegetation and chemical point of view. The community structures of two groups of mites were studied (Acari: Mesostigmata, Oribatida) from a tailing pond, using transect method, in correlation with concentrations of heavy metals (As, Cu, Pb, Ni, Mn and Zn), with abiotic factors (altitude, aspect, soil temperature, soil humidity, soil pH) and biotic factor (vegetation coverage). Taking into account the mite communities, in total, 30 mite species were identified, with 1009 individuals and 18 immatures (10 species with 59 individuals, 5 immatures of Mesostigmata and 20 species with 950 individuals, 13 immatures of Oribatida). The investigated habitats from the tailing pond were grouped in five transects, with different degree of pollution, based on total metal loads. Taking into account of the connection between mites communities, abiotic factors and heavy metals, each transect were characterized through specific relationship. Using multivariate statistical analysis, we revealed that the occurrence of some Oribatida species was strongly correlated with vegetation coverage, soil pH and soil humidity, though concentrations of Cu, As, Mn, Ni and Zn also had an influence. Pb and Zn concentrations were shown to influence the occurrence of Mesostigmata mites. The heterogeneity of mites species richness at 2 m^2^ scale was correlated with a metric related to the heterogeneity of heavy metals at the same scale.

## Introduction

Mining activities have a significant impact on ecosystems, also affecting the vegetation and soil fauna, by removing the topsoil^1,2^. Natural ecosystems are characterised by complex and interrelated trophic interactions, even on the soil level. Soil properties, vegetation and soil fauna are strongly correlated^3,4^. Thus, successful ecological restoration must take account of all these components, as well as the edaphic fauna. Soil fauna participate directly and indirectly in the decomposition process, soil genesis and soil characteristics (porosity, aeration, fertility, infiltration) and are involved in nutrient cycling^2,5–7^. Many studies have revealed that edaphic fauna, whose life cycle is largely within the upper ten centimetres of soil, are significantly affected by the removal and storage of soil^2,4,8^. Ecological restoration of soil invertebrates in post-mining areas takes between 10–20 years^9–11^. Due to their high diversity and their small dimensions, monitoring studies of soil fauna are scarce, even though these invertebrates are useful bioindicators of human disturbance and can be used to define mine-site soil condition and quality^2,11–14^. According to Gerlach *et al*., 2013^15^, invertebrates as bioindicators, may reflect trends in species richness and community composition more accurately than vertebrates^16^. This finding is particularly true in studies of mine site rehabilitation, where invertebrates are also more cost-effective to use. Mesofauna (soil invertebrates with body size between 100 µm-2 mm), are one of the most important groups in soil ecology^7,16,17^. Within the soil mesofauna, mites are often used as bioindicators to investigate the ecological stage of soils from natural or anthropogenic ecosystems. Many studies confirm the value of soil mites as bioindicators, due to their high population density, species richness, sensitivity to soil conditions, to the availability of well-developed sampling methods, and to their ability to accumulate metals^14,18–20^. According to the latest classification, the mites (Acari), can be divided into two superorders: Parasitiformes (with orders: Opilioacarida, Holothyrida, Ixodida and Mesostigmata) and Acariformes (with orders: Trombidiformes and Sarcoptiformes- suborder Oribatida)^21^. Mite species of the Mesostigmata (mainly predators) and Oribatida (decomposers) groups are sensitive to disturbance such as mining activities^22–27^.

In Europe, many studies were performed concerning the spatial patterns and response of the two groups of soil mites (Mesostigmata and Oribatida) to specific environmental conditions in anthropogenic, industrially disturbed soils (*e.g*. spoil areas, tailing ponds, abandoned mining and smelting areas, post-industrial dumps). For instance, in Belgium, Czech Republic, Georgia, Germany, Hungary, Italy, Norway, Poland, Slovakia and Spain^1,9,10,13,18,20,25,26,28–39^. There are scale dependent patterns of species distribution in mining areas^40^ and scale specific processes leading to this distribution^41,42^. These scale specific patterns and processes are influenced by the heterogeneity of the tailing material^43^.

It is known that Romania has one of the biggest reserves of the gold from Europe. During the communist period in Romania (especially 1965–1990), gold and silver exploitation increased drastically. The landscape was modified from natural meadows and forests to anthropogenic ecosystems (*e.g*. mines, spoil areas, tailing ponds). Soil mites from such anthropogenic areas were very poorly studied and only characterised in terms of their structure and dynamics from a few degraded areas *e.g*. the Retezat Mountains^44,45^.

The main objectives of the present research were to establish the pollution level of the mine tailing substrate, using a modern technique of analysis (X-ray fluorescence spectrometry) and, for the first time in Romania, to highlight the impact of such pollution on soil mite communities in such type of anthropic ecosystem. In other words, the study hypotheses were: 1) is the mine tailing characterized by the different levels of heavy metal pollution? 2) which are the specific environmental factors of the investigated area? 3) the mite’s populations are influenced by this pollution and by environmental variables and at what scale?

The present research was focussed on a tailing pond (area: 36 hectares), located in Hunedoara county, Romania, and took into account the degree of pollution and the cumulative influence of heavy metals and of environmental variables (*e.g*. soil temperature, soil humidity, soil acidity, altitude, percent of vegetation cover and exposure).

This study will fill the gap concerning the acarological data correlated with heavy metal soil pollution, from a less studied country, located in the East part of Europe, using modern methods of chemical and statistical analysis.

## Material and Methods

### Study area

**Figure 1:**
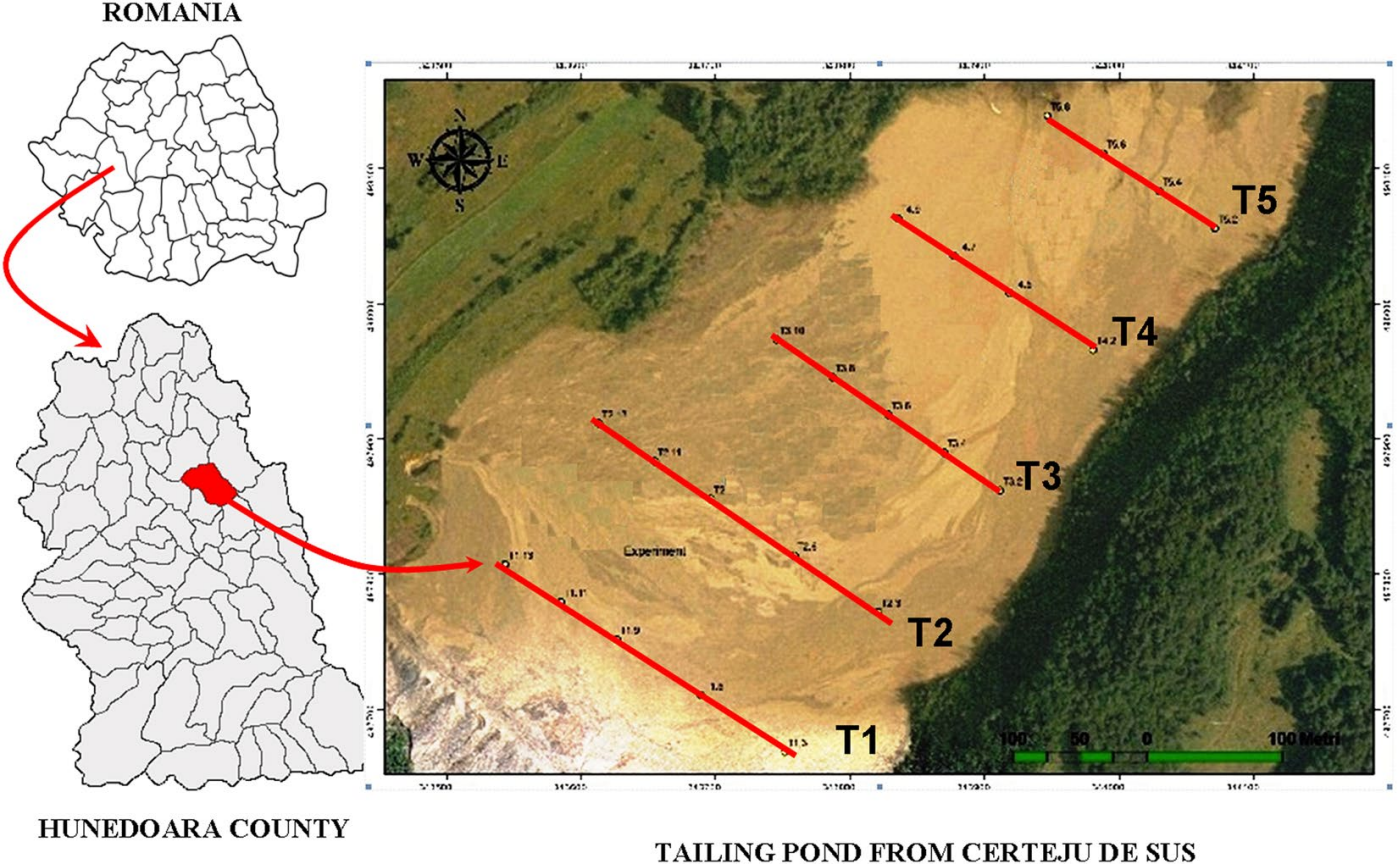
Location of the sampling sites from investigated tailing pond in Certeju de Sus- Hunedoara County, Romania, 2015.

The present study began in 2015, in Hunedoara County, commune Certeju de Sus. Certej mining area is situated in the Metaliferi Mountains, belonging to the Southern Apuseni Mountains in the Western Carpathians of Romania. The small Certej catchment covers an area of 78 square kilometers^46^ (Fig. 1).

According to^47^, the Certej ore deposit is part of the “Golden Quadrilateral” Săcărâmb - Brad - Roşia Montana - Baia de Arieş, a very old mining area (from the pre-Roman era), which produced between 1,000 and 2,000 t of gold. Exploitation of the Certej ore deposit dates from the seventeenth century. From the geological point of view, the Certej ore deposit is classified as an intermediate sulphide epithermal deposit due to the association of Au with Ag, Pb, Zn and, to a lesser extent, with Te and As. The residual material resulted after processing the ore, was transported to waste disposal sites. One of these is the Certej tailing pond, active from 1984 to 2006. The tailing pond is covered with vegetation and its groundwater is monitored^46,48^.

This tailing pond is situated at 45°57′38.97″ N and 22°58′55.39″ E, and its surface area is about 36 hectares. The altitude of the tailing dam varies between 330 and 340 metres. In order to investigate the heterogeneity of the tailing pond and to identify that it could be characterized by the different pollution levels, five transects were established in this ecosystem (T1, T2, T3, T4, T5) (Table 1). The distance between transects is about 25 metres. Due to variation in the width of the tailing pond, the lengths of transects themselves varied between 200 and 325 metres (Fig. 1).

**Table 1:** Averages concentrations of heavy metals (mg/kg^−1^) from the investigated transect, on the tailing pond from Certeju de Sus - Romania, 2015.

Transect	As	Cu	Mn	Ni	Pb	Zn
T1	178.1 (± 21.45)	24.3 (± 2.17)	863.6 (± 49.20)	64.13 (± 19.47)	153.68 (± 49.06)	755.54 (± 269.82)
T2	234.56 (± 56.12)	24.14 (± 15.31)	1054.2 (± 281.65)	68.70 (± 13.73)	159.6 (± 19.58)	1028.54 (± 554.55)
T3	211.04 (± 29.82)	29.72 (± 6.65)	1014.8 (± 202.72)	68.25 (± 25.06)	172.64 (± 34.84)	672.86 (± 255.44)
T4	225.36 (± 19.56)	40.92 (± 6.52)	1304.33 (± 600.56)	33.51 (± 29.05)	236.2 (± 47.287)	608.8 (± 50.68)
T5	268.37 (± 79.57)	42.10 (± 9.05)	1269.75 (± 378.80)	56.07 (± 20.07)	292.35 (± 90.37)	681.15 (± 120.96)
Legal normal values	5	20	900	20	20	100
The alert values	15–25	100–200	1500–2000	75–200	50–250	300–700
The intervention values	25–50	200–500	2500–4000	150–500	100–1000	600–1500

Legend: the normal, alert and intervention values are according to M.O. no. 756/03.11.1997.

Transect T1 was located at 45°57′55.57″ N; 22°59′2.39″ E, on an altitude of 330 meters, west. Its length was by 325 meters. The dominant vegetation was represented by herbaceous, tree and moss layers. Dominant plants species at 0–1 metres height were: *Conyza canadensis* (L.) Cronquist; *Medicago sp*., bryophytes, with a cover by 24%. On 1–5 meters height were: *Calamagrostis arundinacea* (L.); *Phragmites australis* (Cav.) Trin. Ex Steud; *Robinia pseudoacacia* L., with a cover by 72%.

Transect T2 was located at 45°57′52.59″ N; 22°58′58.30″E, on an altitude of 337 meters, west. Its length was by 310 meters. The dominant vegetation was represented by herbaceous and moss layers. Dominant plants species at 0–1 metres height were: *Conyza canadensis* (L.) Cronquist; *Medicago sp., Agrostris sp*., bryophytes, with a cover by 37%. On 1–5 meters height, dominant plant species were: *Calamagrostis arundinacea* (L.); *Robinia pseudoacacia* L., with a cover by 42%.

Transect T3 was located at 45°57′50.90″ N; 22°58′51.27″ E, on an altitude of 337 meters, west. Its length was by 250 meters. The dominant vegetation was represented by herbaceous and moss layers. Dominant plants species at 0–1 metres height were: *Equisetum palustre* L., *Agrostris sp*., bryophytes, with a cover by 21%. On 1–5 meters height dominant plant species were: *Calamagrostis arundinacea* (L.); *Phragmites australis* (Cav.) Trin. Ex Steud, with a cover by 72%.

Transect T4 was located at 45°57′48.34″ N; 22°58′46.20″ E, on an altitude of 338 meters, west. Its length was by 220 meters. The dominant vegetation was represented only by herbaceous layer. Dominant plants species at 0–1 metres height were: *Tussilago farfara* L., bryophytes, with a cover by 12%. On 1–5 meters height dominant plant species were: *Calamagrostis arundinacea* (L.); *Phragmites australis* (Cav.) Trin. Ex Steud, with a cover by 98%.

Transect T5 was located at 45°57′44.92″ N; 22°58′43.09″ E, on an altitude of 340 meters, west. Its length was by 200 meters. The dominant vegetation was represented only by tree layer. Dominant plant species at 0–1 metres height was represented by *Epilobium palustre* L., with a cover by 11.50%. On 1–5 meters height dominant plants were: *Betula pendula* Roth; *Salix purpurea* L.; *Phragmites australis* (Cav.) Trin. Ex Steud, with a cover by 88.75%.

### Soil samples

The soil samples for mite fauna were taken as well from the five transects. They were collected in September 2015, by taking 42 cores per transect to a depth of 10 cm, with a MacFadyen corer (5 cm diameter). In total 210 soil samples were analysed. In each station the cores were sample on a surface of about 2 m^2^. From a total of 210 soil cores, we extracted 18 immatures, 1009 adult individuals, with 30 mite species. We used modified Berlese-Tullgren method of extraction, with funnels (for 14 days on natural light), mites being kept in ethyl alcohol and clarified in lactic acid. The taxonomical identification was made till species level, using published identification keys^49–54^. All species were deposited in the collection of the Stationary Posada of Institute of Biology-Bucharest, Romanian Academy.

The concentrations of six heavy metals were quantified: As - arsenic; Cu– copper; Ni - nickel; Mn- manganese; Pb- lead, Zn- zinc. In order to correlate the heterogeneity of mite fauna distribution in each station with tailings geochemical heterogeneity at a reasonable cost and effort, an number of 210 measurements with field XRF were performed *in situ* on the surface of the tailing dam (0 cm depth), using the same number of replicates in each station on a 2 m^2^ surface as for mites sampling. Five transects were investigated (T1, T2, T3, T4, T5). The chemical analyses were made, using XRF (X-ray fluorescence spectrometry Thermo Scientific Niton GOLDD). The comparison between the accepted legal values (on three levels: references values, alert values and intervention values) and the average heavy metals concentrations from investigated transects are presented in Table 1. Total metal load (TML) were calculated in order both to make a comparison between transects and to classify them. The TML was calculated as the sum of their levels standardised by (x-xmin)/(xmax-xmin), x = concentration of heavy metal^14,55^.

Using formula applied by Santamaría *et al*., 2012 (45), in order to facilitate comparison between transects, three groups of transects were distinguished: T1, T3, T4 the less polluted transect (TML = 25–30); T2 the medium polluted ones (TML = 30–35) and T5 the most pollution transect (TML = 35–40) (Table 2).

**Table 2:** The total metal load (TML) and the degree of pollution (DP) from the investigated transect, on the tailing pond from Certeju de Sus - Romania, 2015.

Transect	As	Cu	Mn	Ni	Pb	Zn	TML	DP
T1	17.31	0.06	<0	0.80	4.46	3.28	25.91	Less polluted
T2	22.95	0.05	0.26	0.89	4.65	4.64	33.44	Medium polluted
T3	20.60	0.12	0.19	0.88	5.09	2.86	29.74	Less polluted
T4	18.90	0.13	0.13	0.09	5.24	1.78	26.28	Less polluted
T5	26.34	0.28	0.62	0.66	9.08	2.91	39.87	Highly polluted

The soil samples were collected in order to quantify some environmental variables, as: soil humidity-Rh%; soil temperature-T; pH – soil acidity. The number of samples was equal as those for soil mites fauna (42 samples/transect). In the same time another variables were quantified: percent of vegetation cover (for two categories of height: from 1–5 metres and from 0–1 metre) - veg.cov., altitude- Alt; exposure- Exp. (W - West; E - East; S - South; N – North). The average values of abiotic factors are presented in Table 3.

**Table 3:** Average values of the abiotic factors from the investigated transects, on the tailing pond from Certeju de Sus – Romania (T - soil temperature; Rh - soil humidity; pH - soil acidity), 2015.

Transect	T (°C)	Rh (%)	pH
T1	20.57 (±1.64)	6.56 (±2.22)	6.68 (±0.11)
T2	20.71 (±1.64)	4.73 (±2.10)	6.63 (±0.15)
T3	22.02 (±2.49)	7.41 (±2.96)	6.64 (±0.16)
T4	20.85 (±1.51)	10.51 (±1.59)	6.6 (±0.17)
T5	21.75 (±1.80)	13.55 (±0.65)	6.38 (±0.08)

### Data analysis

Using one-way analysis of variance (ANOVA), we tested for significant differences among the three *a priori* defined levels of the degree of pollution.

Canonical Correspondence Analysis (CCA) was used to study responses of the mite community to the environmental factors, heavy metals and degree of pollution. We used the permutation procedure (based on 9999 cycles) to test the significance of constraints (environmental variables) in CCA for all eigenvalues^56^. Prior to analysis, we used the log (*x* + 1) transformation where *x* is the species abundance while the environmental variables were Z-transformed.

Taking into consideration the investigated population parameters (numerical abundance and number of species), we used Generalised Linear Mixed Models (GLMM) to test whether these were related to environmental factors, pollution degree, heavy metals concentration and finally to a combination of environmental factors and metals concentration. The relative performance of the models was assessed using a selection technique based on Akaike’s information criterion corrected for sample size (AICc)^57,58^. Models were ranked, and the one with the lowest AICc was used as the reference for calculating the AIC difference (Δi) and the likelihood of a model given the data and model weights (wi). Models within two AIC units of the AICmin were considered competitive and more plausible than others^57^.

The *in situ* measured concentrations of elements have been processed (after log transformation for normalisation in some cases) by principle component analysis, and then the coefficient of variation of the scores on the first two extracted factors (eigenvalues 3.77 and 2.36 accounting together for 61.33% of the data variability) was computed for each station,as well as the metric 100/CV. These synthetic geochemical metrics were then inspected for correlation with the coefficient of variation of mites’ species richness at each station.

Due to the low number of immatures identified for each group, in multivariate statistical analysis we considered only the adults.

The software R version 3.2.1 and Statistica 8.0 were used to perform all analyses^59^. In particular, PCA was completed using the prcomp procedure. Multivariate analysis CCA was performed using the Vegan package^56^ and GLMMs using the nlme package^60^.

## Results

Taking into account the studied heavy metals, we observed that:

- As, Cu and Pb had the highest concentrations in T5, medium in T2 and T4, lowest in T1;
- Mn had the highest concentrations in T4, medium in T2, T3, T5, lowest in T1;
- Ni and Zn had the highest concentrations in T2, medium in T2, T3, T5 and the lowest in T4.

Considering the total metal loads (TML), the highest value was obtained in T5 and the lowest in T1, T3 and T4. In transect T2, we obtained the medium values of this parameter (Table 2).

Comparison of the mean values of metal concentrations in the three pollution-degree categories showed no significant differences for Ni (F[2,85] = 2.049, P = 0.135) but significant differences for As, Cu, Mn, Pb and Zn (ANOVA F[2,85] > 7.62 and thus P ≤ 0.01) (Table 4).

**Table 4:** Mean differences of the metal concentrations and lower and upper 95% confidence intervals (CI) from the Tukey HSD post hoc comparisons among the three levels of pollution degree.

As	Mean difference	95% CI	Upper	P
		Lower		
Low-High	−68.103	−102.159	−34.047	0.000
Medium-High	−76.535	−115.199	−37.871	0.000
Medium-Low	−8.431	−42.951	26.087	0.830
**Cu**				
Low-High	−11.708	−17.475	−5.942	0.000
Medium-High	−25.318	−31.865	−18.771	0.000
Medium-Low	−13.609	−19.455	−7.764	0.000
**Mn**				
Low-High	−236.659	−435.378	−37.939	0.015
Medium-High	−406.422	−632.033	−180.812	0.000
Medium-Low	−169.763	−371.189	31.661	0.116
**Ni**				
Low-High	−8.128	−21.3107	5.052	0.310
Medium-High	2.127	−1.283	17.093	0.939
Medium-Low	10.256	−3.104	23.617	0.166
**Pb**				
Low-High	−109.746	−14.294	−76.543	0.000
Medium-High	−146.404	−184.100	−108.708	0.000
Medium-Low	−36.658	−70.313	−3.003	0.029
**Zn**				
Low-High	−12.850	−212.791	187.089	0.987
Medium-High	300.657	73.660	527.655	0.006
Medium-Low	313.508	110.844	516.172	0.001

If we make a characterisation of transects, analysing abiotic factors, we revealed that:

- T1 is defined by the lowest average value of the soil temperature, the least acid soil and medium humidity;
- T2 is defined by the lowest average of soil humidity, medium values of pH and temperature.
- T3 has recorded the highest average value of soil temperature and medium values of pH and humidity.
- T4 recorded only medium average values.
- T5 has the most acid soil (Table 3).

**Figure 2:**
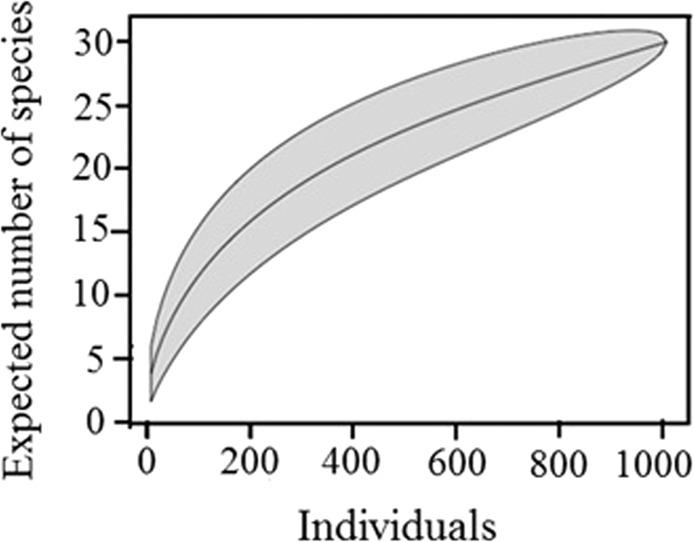
Individual-based accumulation curve for the species richness of mite community. The shaded area represents the 95% confidence intervals.

In total, 30 mite species were identified, with 1009 adult individuals, from two orders: Mesostigmata and Oribatida. If we consider the immature stages, in total 18 specimens were identified. Each investigated transect was described by a characteristic structure of the soil mite communities (species richness, numerical abundance, dominant species) (Table 5). Although 1009 adult individuals from 30 species were observed, the individual-based accumulation curve showed that the number of species was heavily correlated with sampling effort and that the sampling was not sufficient (Fig. 2). The highest abundance of adult individuals was obtained in T5 (406 individuals), whilst T2 held the greatest number of species (14). If we considered the immature stages, the highest number was obtained in T3 (6 specimens) and on opposite is T1 and T5, where only one immature was found.

**Table 5:** Numerical abundance of the mites (Acari: Mesostigmata, Oribatida) identified from investigated transects (T1–T5), 2015.

No.	Species	Code	T1	T2	T3	T4	T5	Total
MESOSTIGMATA								
1	*Amblyseius* sp.	MAmsp	0	1	0	0	1	2
2	*Arctoseius cetratus*	MArce	0	0	0	0	4	4
3	*Asca aphidoides*	MAsap	0	0	1	0	0	1
4	*Asca bicornis*	MAsbi	0	6	5	0	12	23
5	*Hypoaspis cuneifer*	MHycu	0	0	1	0	0	1
6	*Hypoapis praesternalis*	MHypr	0	7	5	4	2	18
7	*Hypoaspis vacua*	MHyva	0	1	0	0	0	1
8	*Leioseius magnanalis*	MLema	0	0	1	0	0	1
9	*Rhodacarellus silesiacus*	MRhsi	0	1	0	0	0	1
10	*Uropoda* sp.	MUrsp	5	0	1	0	1	7
	Total Mesostigmata adults		5	16	14	4	20	59
	Total Mesostigmata immatures		0	0	5	0	0	5
ORIBATIDA								
11	*Ceratozetes fusiger*	OCefu	2	0	0	0	4	6
12	*Ceratozetes gracilis*	OCegr	0	1	0	0	0	1
13	*Cultroribula bicultrata*	OCubi	0	90	68	6	16	180
14	*Diapterobates oblongus*	ODiob	0	6	4	0	0	10
15	*Dissorhina ornata*	ODior	3	0	0	0	0	3
16	*Epilohmannia cylindrica*	OEpcy	1	0	0	0	0	1
17	*Galumna obvia*	OGaob	0	0	0	6	15	21
18	*Lauroppia neerlandica*	OLane	0	106	74	100	344	624
19	*Liacarus coracinus*	OLico	0	0	0	1	0	1
20	*Metabelba pulverulenta*	OMepu	1	0	0	0	0	1
21	*Mycobates carli*	OMyca	0	0	0	1	4	5
22	*Minunthozetes pseudofusiger*	OMyps	0	0	0	0	1	1
23	*Oppia bicarinata*	OOpbi	2	0	0	0	0	2
24	*Oppia concolor*	OOpco	0	0	4	2	0	6
25	*Peloptulus phaenotus*	OPeph	0	1	1	2	0	4
26	*Protoribates lophotrichus*	OPrlo	0	0	0	0	1	1
27	*Sphaerobates gratus*	OSpgr	0	1	0	0	0	1
28	*Sphaerozetes tricuspidatus*	OSptr	0	4	0	0	0	4
29	*Suctobelbella subtrigona*	OSusu	1	1	2	0	0	4
30	*Tectocepheus velatus*	OTeve	1	55	17	0	1	74
	Total Oribatida adults		11	265	170	118	386	950
	Total Oribatida immatures		1	5	1	5	1	13
	Total no. of individuals		16	281	184	122	406	1009
	Total no. of species		8	14	13	8	13	30

Focussing on Mesostigmata mites, in the five transects 10 species were identified, with 59 individuals and 5 immatures. The greatest number of species (6 species) was recorded in T3, whereas only 1 species was seen in T1 and T4 (Table 5). The highest number of individuals was recorded in T5 (20 individuals) and the lowest in T4 (4 individuals). The immatures were identified only in T3 (5 specimens). Dominant species were: *Hypoaspis praesternalis* (23 individuals) and *Asca bicornis* (18 individuals). In contrast, four species were represented by a single individual: *Asca aphidoides, Hypoaspis vacua, Leioseius magnanalis*, and *Rhodacarellus silesiacus*.

Analysing the Oribatida, 30 species were identified, with 950 individuals and 13 immatures. For this group, the most species-rich transect was T2 (9 species), whilst the mite communities in T1, T3 and T4 held just 4 species each. In terms of total adult individuals, the oribatid community in T5 had the highest value (386 individuals), and T1 the lowest, with only 11 individuals. Considering the immature stages, in T2 and T4 they recorded the highest values (5 specimens in each transect) and in T1, T3 and T5 the lowest values (1 specimen) (Table 5). Dominant decomposer mites were: *Lauroppia neerlandica* (624 individuals), *Cultroribula bicultrata* (180 individuals) and *Tectocepheus velatus* (74 individuals). Seven species were represented by single individuals: *Ceratozetes gracilis, Epilohmannia cylindrica, Liacarus coracinus, Metabelba pulverulenta, Minunthozetes pseudofusiger, Protoribates lophotrichus* and *Sphaerobates gratus* (Table 5).

**Figure 3:**
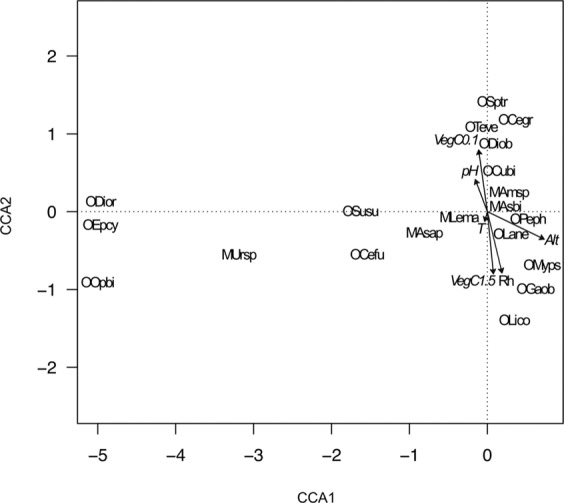
Canonical correspondence analysis bi-plot of the abundance of mite species and environmental factors. Length and direction of arrows indicate the relative importance and direction of change in the environmental variables. Variables are: Alt- altitude, pH – soil acidity, Rh-soil humidity, T – soil temperature, Veg. cov. – percent of vegetation cover (for two categories of height: from 1–5 metres and from 0–1 metre). Species names were abbreviated using the initials of the order and species name (abbreviations are explained in Table 5).

CCA of the association between mite abundance and the environmental factors is shown in Fig. 3. The first two canonical axes accounted for 73.33% (CCA1 = 47.75%; CCA2 = 25.58%) of the total variation in the original matrix. The first canonical axis was highly correlated with altitude (0.86), whereas the second canonical axis was correlated with vegetation cover of 1–5 m height (−0.94) or of 0–1 m height (−0.93) and Rh (−0.92). In the biplot *Asca bicornis*, *Peloptulus phaenotus, Lauroppia neerlandica* were correlated with low altitude, *Tectocepheus velatus* and *Diapterobates oblongus* with high vegetation cover of 0–1 m height, *Cultroribula bicultrata* with high pH and *Galumna obvia* with high vegetation cover ofy 1–5 m height and Rh% (Fig. 3).

**Figure 4:**
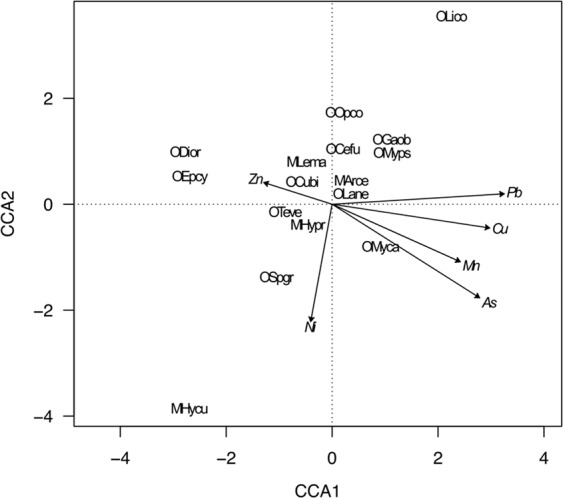
Canonical correspondence analysis bi-plot of the abundance of mite species and heavy metals. Variables are: As - arsenic; Cu - copper; Ni - nickel; Mn - manganese; Pb - lead and Zn - zinc. Other legend as in Fig. 3.

The CCA of the association between mite abundance and the heavy metals is shown in Fig. 4. The first two canonical axes accounted for 69.03% (CCA1 = 50.67%; CCA2 = 18.36%) of the total variation in the original matrix. The first canonical axis was highly correlated with Pb (0.81), Cu (0.74), As (0.70) and Mn (0.61) whereas the second canonical axis was correlated with Ni (−0.55). Species *Sphaerozetes tricuspidatus* from the lower left quadrant was associated with Ni. The right lower quadrant contains the species *Lauroppia neerlandica* and *Mycobates carli* associated with Cu, As and Mn. Species associated with Zn were placed in the upper left quadrant (*Cultroribula bicultrata, Dissorhina ornata, Epilohmannia cylindrica, Leioseius magnanalis*) and species associated with Pb in the upper right quadrant (*Arctoseius cetratus*) (Fig. 4).

Based on model selection, using AIC, models including heavy metals were best supported from both abundance and species richness (Table 6).

**Table 6:** Akaike statistics for model including the species richness and the species abundance.

Model	Covariates	LL	K	AICc	ΔAICc	*w*_i_
*Species richness*						
1	Environmental factors and heavy metals	−142.79	15	315.59	7.51	0.02
2	Environmental factors	−148.39	9	314.79	6.71	0.03
**3**	**Heavy metals**	**−145.04**	**9**	**308.08**	**0.00**	**0.81**
4	Pollution degree	−150.75	5	311.50	3.42	0.15
*Abundance*						
1	Environmental factors and heavy metals	−371.7	15	773.43	7.12	0.03
2	Environmental factors	−392.46	9	802.92	36.60	0.00
**3**	**Heavy metals**	**−374.16**	**9**	**766.32**	**0.00**	**0.97**
4	Pollution degree	−392.88	5	795.77	29.45	0.00

AIC (Akaike’s Information Criterion) differences (ΔAICc) and Akaike weights (wi) were used to rank models relative to the best model (minimum AIC). K – number of parameters; LL – log likelihood.

**Figure 5:**
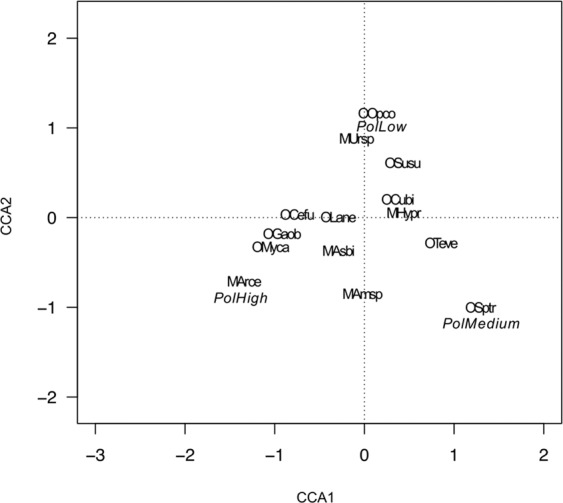
Canonical correspondence analysis bi-plot of the abundance mite species and degree of pollution (Pol): low, medium and high. Other legend as in Fig. 3.

The CCA of the association between mite abundance and the degree of pollution is shown in Fig. 5. The first two canonical axes accounted for 100% (CCA1 = 70.44%; CCA2 = 29.56%) of the total variation in the original matrix. Species from the upper left and right quadrants were associated low levels of pollution: *Suctobelbella subtrigona, Cultroribula bicultrata, Oppia concolor* and *Hypoaspis praesternalis*. The right lower quadrate contains species associated with medium levels of pollution (*Tectocepheus velatus* and *Sphaerozetes tricuspidatus*) and the species associated with high level of pollution were placed in the lower left quadrant (*Lauroppia neerlandica, Galumna obvia, Mycobates carli, Asca bicornis* and *Arctoseius cetratus*) (Fig. 5).

**Figure 6:**
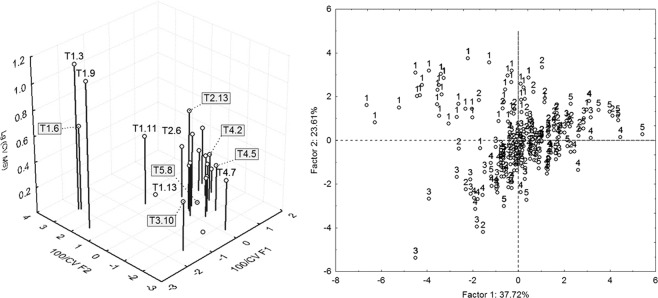
Left: Correlation between the coefficient of variation of mites’ species richness (log transformed) and the metric 100/CVs of the scores extracted by PCA from *in situ* measurements of geochemical variables on the tailing surface in each station with a plot of 2 m^2^. Codes indicate examples of stations on the transects (e.g. T1.3 = transect 1 station 3); Right: byplot of the samples scores on the factors 1 and 2 extracted by PCA from *in situ* geochemical measurements. Numbers indicate that the measurements belong to the same transect, from 1 to 5.

The coefficient of variations of species richness in each station (log transformed) was not correlated with the coefficients of variation (CVs), but was correlated with 100/CVs of the scores of *in situ* geochemical measurements extracted by PCA on the first two factors (Fig. 6). Both correlations with the CVs computed for the first (R = −0.63) and second (R = 0.52). PCA factors were statistically significant (p < 0.05).

## Discussion

The measured concentrations of heavy metals from the five transects all exceeded the reference values set in the national legislation^61^ except for Mn in T1, which was lower than the reference values. These results are much higher than those obtained in grasslands within the Zlatna depression of Transylvania, where the pollution of soil was due to the heavy metal atmospheric deposits from an industrial basket of an old mining exploitation factory^14^. If we referred at the maximum concentrations of heavy metals obtained in the studied transects, the As exceed till 54 times than legal normal values, Pb 15 times more, Zn 10 times, Cu double and Ni three times more (Table 1).

This phenomenon is due to the particular characteristics of a tailing pond: there are always metals and metalloids, because no extraction process is ever 100% efficient. Although there is no universally accepted protocol for directing which trace elements are measured in tailings studies, As, Cu, Pb and Zn are normally quantified and generally have high concentrations^62^.

Considering the pollution level, heavy metal concentrations were highest in transect T5 and lowest in transects T1, T3 and T4. The most highly-polluted transects were characterised by the greatest soil humidity (13.55%), a moderate soil temperature and the most acid soil (6.38)^63^. In contrast, less-polluted transects were characterised by the lowest soil temperature (20.71 °C), medium soil humidity and soil pH values of 6.6–6.68. The lowest soil humidity (4.73%) was recorded in medium-polluted transects.

From soil in the investigated transects, 30 mite species were identified, 10 species (59 individuals, 5 immatures) from the Mesostigmata order and 20 species (950 individuals, 13 immatures) from Oribatida order. For the Mesostigmata, the number of species and numerical abundance recorded on the tailing pond from Certeju de Sus are comparable with those obtained in industrial disturbed soils from Norway (9 species), coal mine dumps from Poland (29–31 species, with 1292–2095 individuals/sq.m.), Spain (2636–3954 individuals/sq.m.), old mine galleries from Poland (28–32 species; with 712–1140 individuals), Germany (12 species with 3043 individuals), Belgium (11 species, with 57 individuals), spoil areas from Romania (6–9 species, with 11–22 individuals)^10,20,36,38,39,45,64–66^. However, if we compare the present results with those obtained in afforested post-industrial habitats from Poland (45 species and 1026 individuals)^35^ the totals for Certeju de Sus are much lower.

Examining the oribatid species and their numerical abundances at Certeju de Sus show comparable results with those obtained in abandoned mining areas, post-industrial dumps, spoil areas, mine galleries from Georgia (14–26 species), Italy (34 species with 8721 individuals), Czech Republic (38 species, with 9034 individuals from 5 sites), Norway (2–22 species), Poland (50 species with 2936 individuals from 10 sites or 1–29 species from four sites) and Romania (6–21 species, with 200–500 individuals)^25,31,33,34,37,38,44^. In afforested dumps, studies in Germany found a higher number of oribatid species (63 species), whilst research on mine galleries in Belgium identified only one species (22 individuals) and in Spain (47 species from 12 sites)^9,10,36^.

Comparing species richness and numerical abundance for Mesostigmata and Oribatida showed markedly higher values for the oribatids at Certeju de Sus. Indeed, the number of oribatid species recorded was twice that of the Mesostigmata and the number of individuals over 16 times greater. These results agree with those obtained from other heavy metal polluted grasslands in Romania or from coal mining areas in Germany or Spain, where the mesostigmatids represented between 5% and 14% and oribatids between 71% and 75% from the total number of mites recorded^1,10,66^.

On the other hand the number of immatures is very low in comparison with the adults. They represent only 1.76% from the total mite abundance (1.36% for Oribatida and 8.47% for Mesostigmata). It is possible that the high concentrations of heavy metals could influence the biological cycle of these soil mites.

We also observed natural rehabilitation of the Certeju de Sus tailing pond (due to the spontaneous vegetation installed here), which involves an ecological succession process^40^. In this context, oribatid fauna may dominate among mites at habitats with initial plant vegetation. Oribatid mites are known to predominate over other groups of mites and mesofauna in most soils^1,67^. The pool of oribatid species, which are capable of performing the role of colonists, is broad^34^. Decomposer oribatids, such as *Lauroppia neerlandica* and *Sphaerozetes tricuspidatus*, are considered pioneer species^34,68,69^.

Even though the degree of pollution was highest in T5, the total number of species and numerical abundance reached the highest values, due to the highest soil humidity and acidity, which in turn provides more favourable habitat for soil mites, especially oribatids (*e.g. Cultroribula bicultrata, Galumna obvia* or *Lauroppia neerlandica*). These results were demonstrated by canonical correspondence analysis of the association between mite abundance and the environmental factors. Specialist studies have revealed that, in heavy metal polluted soils, the number of bacteria, Actinomycetes and fungi does not decrease, suggesting that such microorganisms may resist increased or even toxic concentrations of heavy metals, and thus constitute a food source for decomposer oribatids^27,70^. On the other hand, soil pH and humidity are correlated with vegetation cover, affecting local habitat conditions for *C. bicultrata, G. obvia* and *L. neerlandica*, as well as for *Tectocepheus velatus* and *Diapterobates oblongus*^71^. The micro-heterogeneity of the tailing material geochemistry might also play a role in the control of general patterns of mites distribution by reflecting micro-niches (either directly or by correlation with mineralogy, for instance) as suggested by the correlations found between the variability of species richness and that of tailings geochemistry at small scale. However, this remains to be proved in future studies by detailed chemical analysis of each soil samples from which the mites will be extracted (not done here for reasons of costs).

Multivariate analysis at larger, tailing dam scale, revealed that both oribatid and mesostigmatid communities were influenced by the heavy metal concentrations. For example, we observed that *Sphaerozetes tricuspidatus* was influenced by Ni, while *Lauroppia neerlandica* and *Mycobates carli* were influenced by Cu, As and Mn concentrations. Three oribatids (*Cultroribula bicultrata, Dissorhina ornata, Epilohmannia cylindrica*) and the mesostigmatid *Leioseius magnanalis* were associated with Zn concentration, while *Arctoseius cetratus* was influenced by Pb. The canonical correspondence analysis bi-plot of the abundance of mite species and degree of pollution indicated that some mesostigmatid species were characteristic of highly-polluted transects (*e.g. Arctoseius cetratus* and *Asca bicornis*) as were certain oribatids (*Lauroppia neerlandica, Mycobates carli*, *Galumna obvia* and *Ceratozetes fusiger*). In terms of numerical abundance, *Asca bicornis* and *Lauroppia neerlandica* were the most abundant species for the tailing dam ecosystem.

The mesostigmatid species *Asca bicornis* has also been found in polluted areas in the first stage of succession in derelict industrial lands spoil heap, urban ecosystems and in heavy metal polluted grasslands^14,39,54,72^. *Arctoseius cetratus* is found in industrial wastelands, slag heaps and spoil areas, having a great ability to colonise new environments (through phoresy) and is therefore numerous in early stage succession. *A. cetratus* has a high reproduction rate, short development time, tolerance to the chemical contamination of soil and a negative response to Sulphur compounds originating from air pollution^54,72^.

Within the oribatid fauna, we found some species (*e.g. Lauroppia neerlandica*) in the highly-polluted transects, which were influenced by concentrations of heavy metals. Other researchers have shown *L. neerlandica* to be a pioneer-dominant species in post-industrial dumps in Norway, while *Galumnia obvia*, which is resistant to heavy-metal pollution, was observed very close to a metallurgical plant in Russia, in post-industrial dumps in Polan and in airborne polluted urban parks in Romania^72–75^. In contrast, the ubiquitous species, *Mycobates carli*, which has a strong affinity for lichen- and moss-rich habitats, represents the first stages of a natural ecological succession from a tailing pond^76,77^.

Another pioneer species for the process of ecological regeneration, inhabiting the moss layer from peat-bogs or forest soil, is *Sphaerozetes tricuspidatus*^68,69^. This oribatid, together with *Tectocepheus velatus*, was found in less-polluted transects at the Certeju de Sus tailing pond. According to^37^, *T. velatus* is a unique oribatid species, with the ability to live in a broad range of environments from forests to bare lands and often dominates various habitats. This species was found to be able to persist in a wide range of heavy metal pollution. It was identified in soil from: (a) metallurgical plant in Russia; (b) abandoned mines and smelting areas in Italy; (c) planted mines in Spain; (d) heavy metal-polluted grasslands in Poland or forests from Romania; (e) spoil heaps in Czech Republic; (f) post-industrial dumps in Norway; and (g) polluted urban parks in Romania^10,18,31,33,34,72,73,75,78^. Two abilities of *T. velatus* are important to its abundance in such polluted sites: (1) to accumulate a moderate amount of heavy metals compared with other oribatid species; or (2) to inhabit lichens (*e.g*. the thalli of *Cladonia rei*) from post-smelting dumps, which are much less contaminated than the substrate of the dump itself^37^. Other studies revealed that *T. velatus* was indifferent to metal concentrations in its body. The body burden of Cd, Cu and Zn in *T. velatus* increased with the increasing concentrations of these metals in soil^75^. Another mesostigmatid species present in less-polluted transects was *Hypoaspis praesternalis*. This species survives in anthropogenic ecosystems, being dominant in heavy metal polluted grasslands or urban parks in Romania, but also in reforested plots in Poland^14,39,72,79^.

*Suctobellba subtrigona* and *Oppia concolor* were found in medium polluted transects from the tailing pond. The first one has also been noted in forests polluted with heavy metals and fluorine, while the second was identified in soil from restored mines in Spain^10,78^.

## Conclusions

Soil mite communities (Acari: Mesostigmata, Oribatida) and environmental variables (altitude, soil acidity, humidity, temperature, vegetation coverage and heavy metals concentrations) from a tailing dam were studied, using transects methods. The considered population parameters were species richness, species composition and numerical abundance. Taking into account the total metal load, the five investigated transects were classified in three categories: most polluted, medium and less polluted. All studied heavy metals (As, Cu, Mn, Ni, Pb, Zn), recorded very high concentrations (between double and 54 times more than legal normal values). Considering the environmental variables, the tailing dam was characterized by the specifically conditions, for each studied transects. So, the first and the second hypothesis of this study were confimed. With respect to the third one, we observed that the mites’ communities (Mesostigmata and Oribatida) and abiotic factors from most polluted transects recorded clearly distinguishable patterns from those in the less polluted ones. This study demonstrated that heavy metal pollution and the characteristic environmental conditions of a tailing pond had a great impact on the community structure of soil mites. Oribatida species were influenced by the vegetation coverage, soil pH and soil humidity, and by the concentrations of Cu, As, Mn, Ni and Zn. Mesostigmata mites were influenced by the Pb and Zn concentrations. A metric related to the heterogeneity of the geochemistry also played a role on the distribution at small, 2 m^2^ scale.

Taken together these studies into the mite fauna and communities of polluted ecosystems from Romania, we demonstrate both the impact of pollution on community structure and potentially the value of mites in measuring the success of site restoration and amelioration. From a basic research perspective, tailing dams provide opportunities for studies comparing the successional patterns of distributions of organisms with different scales, like mites and plants, in relation to the heterogeneity of environmental variables at their specific scales.

## Data Availability

The data sets analysed during the current study are available from the corresponding author on reasonable request.
